# The pelvic vascular injury score (P-VIS): a prehospital instrument to detect significant vascular injury in pelvic fractures

**DOI:** 10.1007/s00068-023-02374-x

**Published:** 2023-10-23

**Authors:** Christopher Spering, Wolfgang Lehmann, Stefanie Möller, Dan Bieler, Uwe Schweigkofler, Lisa Hackenberg, Stephan Sehmisch, Rolf Lefering, TraumaRegister DGU

**Affiliations:** 1 Department of Trauma Surgery, Orthopaedics and Plastic Surgery, Goettingen University Medical Center, Universitaetsmedizin Goettingen, Robert-Koch-Strasse 40, 37075 Göttingen, Germany; 2 Department of Orthopedic Trauma Surgery, BG Unfallklinik Frankfurt am Main, Frankfurt am Main, Germany; 3 Department of Orthopaedics and Trauma Surgery, Heinrich Heine University Medical School, Düsseldorf, Germany; 4 Department for Trauma Surgery and Orthopaedics, Reconstructive Surgery, Hand Surgery, Burn Medicine, German Armed Forces Central Hospital Koblenz, Koblenz, Germany; 5 Department of Trauma Surgery, Hannover Medical School (MHH), Hannover, Germany; 6 Institute for Research in Operative Medicine (IFOM), University of Witten/Herdecke, Cologne, Germany; 7 Committee on Emergency Medicine, Intensive Care and Trauma Management (Sektion NIS) of the German Trauma Society (DGU), Cologne, Germany

**Keywords:** Peripelvic vascular injury, Pelvic bleeding, Prehospital score, Control bleeding, Pelvic injury

## Abstract

**Purpose:**

The purpose of this study was to identify predictive factors for peri-pelvic vascular injury in patients with pelvic fractures and to incorporate these factors into a pelvic vascular injury score (P-VIS) to detect severe bleeding during the prehospital trauma management.

**Methods:**

To identify potential predictive factors, data were taken (1) of a Level I Trauma Centre with 467 patients (ISS ≥ 16 and AIS_Pelvis_ ≥ 3). Analysis including patient’s charts and digital recordings, radiographical diagnostics, mechanism and pattern of injury as well as the vascular bleeding source was performed. Statistical analysis was performed descriptively and through inference statistical calculation. To further analyse the predictive factors and finally develop the score, a 10-year time period (2012–2021) of (2) the TraumaRegister DGU^®^ (TR-DGU) was used in a second step. Relevant peri-pelvic bleeding in patients with AIS_Pelvis_ ≥ 3 (*N* = 9227) was defined as a combination of the following entities (target group PVI_TR-DGU_ *N* = 2090; 22.7%): pelvic fracture with significant bleeding (> 20% of blood volume), Injury of the iliac or femoral artery or blood transfusion of ≥ 6 units (pRBC) prior to ICU admission. The multivariate analysis revealed nine items that constitute the pelvic vascular injury score (P-VIS).

**Results:**

In study (1), 467 blunt pelvic trauma patients were included of which 24 (PVI) were presented with significant vascular injury (PVI, *N* = 24; control (C, *N* = 443). Patients with pelvic fractures and vascular injury showed a higher ISS, lower haemoglobin at admission and lower blood pressure. Their mortality rate was higher (PVI: 17.4%, C: 10.3%). In the defining and validating process of the score within the TR-DGU, 9227 patients met the inclusion criteria. 2090 patients showed significant peripelvic vascular injury (PVI_TR-DGU_), the remaining 7137 formed the control group (C_TR-DGU_). Nine predictive parameters for peripelvic vascular injury constituted the peripelvic vascular injury score (P-VIS): age ≥ 70 years, high-energy-trauma, penetrating trauma/open pelvic injury, shock index ≥ 1, cardio-pulmonary-resuscitation (CPR), substitution of > 1 l fluid, intubation, necessity of catecholamine substitution, remaining shock (≤ 90 mmHg) under therapy. The multi-dimensional scoring system leads to an ordinal scaled rating according to the probability of the presence of a vascular injury. A score of ≥ 3 points described the peripelvic vascular injury as probable, a result of ≥ 6 points identified a most likely vascular injury and a score of 9 points identified an apparent peripelvic vascular injury. Reapplying this score to the study population a median score of 5 points (range 3–8) (PVI) and a median score of 2 points (range 0–3) (C) (*p* < 0.001). The OR for peripelvic vascular injury was 24.3 for the patients who scored > 3 points vs. ≤ 2 points. The TR-DGU data set verified these findings (median of 2 points in C_TR-DGU_ vs. median of 3 points with in PVI_TR-DGU_).

**Conclusion:**

The pelvic vascular injury score (P-VIS) allows an initial risk assessment for the presence of a vascular injury in patients with unstable pelvic injury. Thus, the management of these patients can be positively influenced at a very early stage, prehospital resuscitation performed safely targeted and further resources can be activated in the final treating Trauma Centre.

## Introduction

Severe trauma still is one of the leading causes of death in Western countries and especially in people below 40 years of age [8]. 25% of severely injured patients show significant pelvic fractures (Abbreviated Injury Scale, AIS ≥ 3) [5, 7]. 3–9% of them are in hemodynamically unstable condition due to concomitant vascular injury [2, 3, 5, 10, 13, 15]. Mortality rates of severely injured patients with mechanically unstable pelvic fractures go up to 18–20% [5, 11] and hemodynamically relevant pelvic vascular injuries boost mortality rates up to levels as high as 33–40% [10, 19]. Whenever high energy is transferred to the human body causing severe injury, rapid damage control and specific individualised treatment are needed [1, 4, 6, 16, 17, 19, 20]. But different patterns of injury and individual capacities of compensation do not make it easy for trauma teams in the prehospital trauma management and in the early clinical assessment to obtain fast, conclusive and complete injury assessment [16, 24, 25]. Time to control the bleeding is especially important in severely injured patients with pelvic vascular injury due to a rapid loss of high blood volume into the pelvis and pelvic soft tissue compartments without clinical signs other than a haemodynamic instability [17]. Bleeding of arteries or venous plexus is life-threatening and reduces time for decision-making and treatment and associated with elevated mortality rates and lower outcome level [13, 16]. Thus, time is of the essence in patients with unstable pelvic injury and peripelvic vascular injury [3–5, 7, 9, 10, 12, 13, 16]. It is important to recognise those patients who are in need of a pelvic binder, application of tranexamic acid, calculated resuscitation and a rapid transport to a maximum care unit. The capacity of massive transfusion, interventional bleeding control including angio-embolization and surgical expertise in managing such severe injuries as fast as possible, needs to be organised during the prehospital trauma management already [19–21, 23–25].

Bleeding in complex pelvic trauma is in 10–20% caused by arterial vascular injury only. More often it is caused by venous vascular injury (80–90%) i.e. ventral sacral plexus with haemorrhagic shock, making it even harder to detect and treat [3, 10, 12, 15].

The purpose of this retrospective cohort study was to develop a score to detect peripelvic vascular injury in major trauma patients with pelvic fractures at an early stage in the prehospital trauma management.

## Methods

### Local data analysis

Our Level I Trauma Centre is a 1500-bed institution, which receives an average of 900 severe trauma patients through the Trauma Resuscitation Unit (TRU) each year. All patients admitted to the TRU were suspected of severe trauma, according to the regional triage system. The standard trauma care in the hospital is in concordance with the Advanced Trauma Life Support^®^ (ATLS^®^) protocol and the recommendations of the German Level 3 guideline on the treatment of patients with severe injury [4, 17]. It includes an initial survey with imaging using extended Focused Assessment with Sonography in Trauma (eFAST), resuscitation and a Whole-Body CT Scan (WBCT) for complete injury assessment. A radiologist and a vascular surgeon are part of the trauma team, whilst a radiologist with interventional expertise is on call.

A retrospective analysis of all severely injured (ISS ≥ 16) primary admissions within a 5-year time period to the TRU with significant pelvic injury (AIS ≥ 3) identified 467 cases.

In the initial data analysis of all patient’s charts and digital recordings including radiographical diagnostics, mechanism and pattern of injury were identified and classified as well as the specific pelvic vascular bleeding source. Every case with vascular involvement and pelvic fracture was then analysed for age, sex, blunt or penetrating trauma, hospital stay, intensive care unit (ICU) stay, number of operations, method of treatment, mortality rate, trauma scores, prehospital fluid management and initial laboratory results as well as prehospital and clinical management strategies.

The cohort was divided into patients with vascular injury (PVI, *N* = 24) and control (C, *N* = 443). Statistical analysis was performed descriptively and through inference statistical calculation using univariate analysis and Mann–Whitney *U* test. Significance was defined as a *p* value *p* ≤ 0.05.

### Trauma registry analysis

In a second analysis, surrogate parameters which may suspect a vascular involvement in major trauma patients were identified. Prehospital assessed parameters were analysed as well as parameters from the early management in the TRU, to identify and characterise vascular injury in pelvic fractures in a prehospital setting.

To further define and validate the score, the data set of a 10-year time period of the TraumaRegister DGU^®^ (TR-DGU) of the German Trauma Society (Deutsche Gesellschaft für Unfallchirurgie, DGU) was used. The TR-DGU was founded in 1993 [22]. The aim of this multi-centre database is the pseudonymised and standardised documentation of severely injured patients. Participation in TR-DGU is voluntary. For hospitals associated with the TraumaNetzwerk DGU^®^ (TNW), the entry of at least a basic data set is obligatory for reasons of quality assurance. Currently, approximately 30,000 cases (basic group of patients) from more than 650 hospitals are entered into the database per year.

Data are collected prospectively in four consecutive time phases from the site of the accident until discharge from hospital: (A) prehospital phase, (B) emergency/resuscitation room and initial surgery, (C) intensive care unit, and (D) discharge. Documentation includes detailed information on demographics, injury patterns, comorbidities, pre- and in-hospital management, course on intensive care unit, relevant laboratory findings including transfusion data, and outcome. Included are patients who are admitted to hospital via the resuscitation room and subsequently receive intensive or intermediate care and patients who arrive at hospital with vital signs and die before admission to the intensive care unit. The infrastructure for documentation, data management, and data analysis is provided by the AUC—Academy for Trauma Surgery (AUC—Akademie der Unfallchirurgie GmbH (AUC), which is affiliated with the DGU. Scientific leadership is provided by the Committee on Emergency Medicine, Intensive Care and Trauma Management (Sektion NIS) of the DGU. Participating hospitals submit their pseudonymised data to a central database via a web-based application. Scientific data analysis is approved according to a peer review procedure established by Sektion NIS. This study is in accordance with the publication guideline of the TR-DGU and is registered under the TR-DGU Project-ID 2022-016.

Based on the local results, potential predictors from the pre-hospital phase were analysed one by one and in a multivariate model. It was intended to create a simple point score where each additional point increases the risk of vascular injury.

The data sets of 10 years (2012–2021, 385,388 patients) of the TR-DGU were used to define the final score within a study group of 9227 patients who had been diagnosed unstable pelvic fracture (AIS ≥ 3).

Statistics were made with SPSS^®^ (Version 28, IBM Inc., Armonk, NY, USA). Descriptive analysis was performed with counts and percentages for categorical variables, and mean with standard deviation (SD) for continuous measurements. In case of considerably skewed data, median and interquartile range (IQR) were provided in addition. Significance was defined as a *p* value < 0.05 using the Chi-squared test and Mann–Whitney *U* test for metric and ordinal characteristics. Outcome and prognosis parameters were calculated and put into relation to the risk of death estimation (RISC II score) [14]. Multivariate logistic regression analysis was performed with PVI as dependent variable. Results were presented as odds ratios (OR) with 95% confidence intervals (CI-95).

**Fig. 1:**
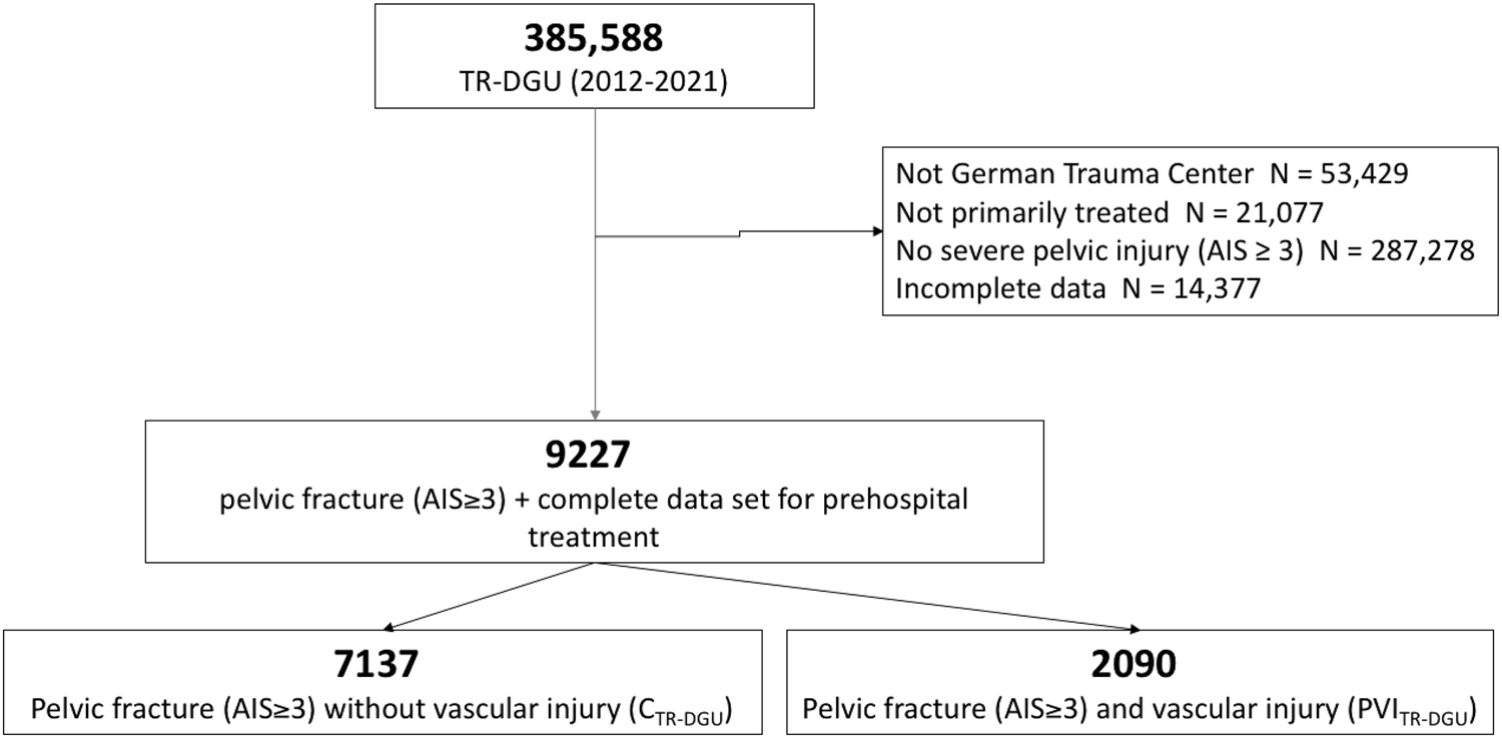
Flow chart showing the included patients of the Trauma Register DGU^®^. (PVI_TR-DGU_—pelvic fracture and vascular injury; C_TR-DGU_—control cohort with a pelvic fracture without vascular injury. *AIS* Abbreviated Injury Scale

The study group included 9227 patients, primarily admitted to a German hospital with serious pelvic fracture (AIS ≥ 3) and complete prehospital data (see Fig. 1).

Patients with relevant peripelvic bleeding (PVI_TR-DGU_ group) were identified by the combination of the following three entities:

- Pelvic fracture with significant bleeding (> 20% of blood volume): AIS-Codes 856,173.5, 856,164.5
- Injury of the iliac or femoral artery: AIS-Codes 820,208.4, 820,299.3
- Blood transfusion of ≥ 6 units of packed red blood cells (pRBC) prior to ICU admission

## Results

**Fig. 2:**
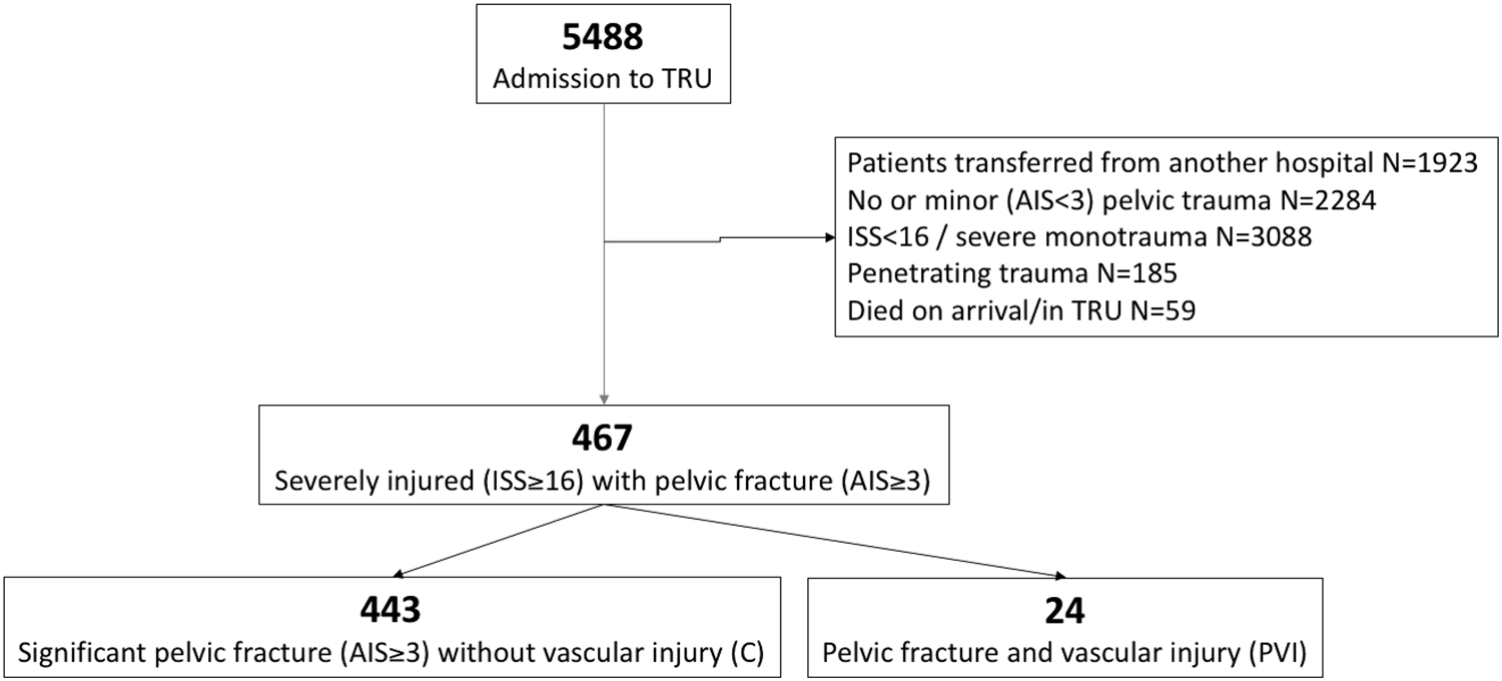
Flow chart showing the included patients within the Level I Trauma Centre. (PVI—pelvic fracture and vascular injury; C—control cohort with a pelvic fracture without vascular injury. *TRU* Trauma Resuscitation Unit, *AIS* Abbreviated Injury Scale, *ISS* Injury Severity Score

In the defined time period, 467 blunt pelvic trauma patients (ISS ≥ 16 and AIS_Pelvis_ ≥ 3) met the inclusion criteria of our Level I Trauma Centre, of which 24 patients (5.1%) were diagnosed relevant vascular injury (PVI) (Fig. 2).

Within the time period of 10 years (2012–2021) of the TR-DGU, 9227 patients with complete prehospital documentation and pelvic fracture (AIS_Pelvis_ ≥ 3) met the inclusion criteria (22.7% with peripelvic vascular injury (PVI_TR-DGU_).

### Epidemiology

Demographics, clinical characteristics and outcome of the patients of the local Level I Trauma Centre are listed in Table 1, showing significant differences in mortality rate and a clear tendency towards a higher percentage of male patients, younger age, high energy trauma, higher Injury Severity Score (ISS) and additional injury to the central nervous system (CNS), peripheral nerve system (PNS), abdomen, bladder and genitals in PVI. The anatomical localization of the vascular injury in the PVI group (majority showed injury to iliac vessels, mesenteric vessels and/or pre-sacral venous plexus) is shown in Table 2. The results of the TR-DGU show, that 7.6% (23,604 of 310,882 patients) of the severely injured suffered an unstable pelvic fracture (AIS_pelvis_ ≥ 3). Within the study collective, 22.7% (2090 of 9227 patients) showed significant bleeding due to a peri-pelvic vascular injury (PVI_TR-DGU_).

**Table 1:** Epidemiological characteristics of control and PVI. Also shown are the *p* values of the *t* test and Kolmogorov–Smirnov test (significance defined as *p* value < 0.05)

	Control (*N* = 433)	PVI (*N* = 24)	*p* value
Male (%)	56.5	75	0.08
Age (years)	46	37	0.14
Mechanism of injury (%)			
Car passenger	41	33	
Motorcycle rider	17	25	
Pedestrian	11	13	
Fall > 3 m/suicide	28	21	
Hit/strike	3	8	
ISS (points)	28	36	0.13
Additional injury (%)			
CNS	43	50	
PNS	21	38	
Thorax/lungs	51	50	
Abdomen	54	68	
Bladder	8	13	
Genitals	24	38	
Mortality rate (%)	10.3	17.4	0.03

**Table 2:** Anatomical localization of vascular injury in the PVI group

Injured vessel	PVI (*N*)
Aorta	1
Vena cava	1
Lumbal vessels	2
Renal vessels	3
Femoral artery	4
Presacral venous plexus	4
Iliac vessels	7
Mesenteric vessels	7

### Therapy

The use of catecholamines due to haemodynamic instability was performed in the prehospital trauma management in only 48% of the PVI patients although they showed a haemoglobin value (Hb) of 8.2 g/dl at arrival in the TRU. 58% of those patients who had received catecholamines in the prehospital setting were still in shock at arrival in the TRU. Due to severe bleeding, all PVI patients received a median of 20 units of transfused pRBC during the initial TRU management. 75% of PVI showed coagulopathy and received a median of 13 fresh frozen plasma units as well as 3 thrombocytes units per patients.

As shown in Table 3, surgical procedures were undertaken significantly more often in patients with peripelvic vascular injuries in the study group of the local Level I Trauma Centre. Open repair of the vascular injury was performed in 71% whilst endovascular procedures had been applied in 26% of the cases. Packing of the pelvis was necessary in 21% of the cases to control the bleeding before the patient was stable enough to undergo a vascular repair. In comparison to the control cohort, osteosynthesis at the day of admission was less often performed in PVI whilst external fixation only or as an additional support was installed more often. The overall duration in hospital as well as ICU therapy was significantly longer in PVI.

**Table 3:** Comparison of PVI and control towards therapy in controlling the bleeding and management of the pelvic fracture as well as ICU therapy and overall duration of admission

	Control (*N* = 433)	PVI (*N* = 24)
Direct open vascular repair	–	71%
Endovascular repair	–	26%
Open tamponade/packing	–	21%
Osteo-synthesis of the pelvis on day of admission	64%	54%
External fixateur only	36%	43%
Additive ext. fixateur	22%	33%
Duration of hospital stay	31.2 days	50.4 days
Duration of ICU therapy	9.8 days	15.3 days
Median number of surgical procedures	2	6

Although patients obviously showed a mechanically unstable pelvic fracture and must have shown some kind of hemodynamical instability, only 25% of the PVI patients had received a pelvic binder (all of the patients who later died in hospital had received a pelvic binder) (Table 4).

**Table 4:** Comparison of survivor and non-survivor amongst 24 patients with PVI regarding epidemiological and hemodynamically relevant parameters as well as prehospital management aspects (mean values)

	PVI (died) *N* = 4	PVI (survived) *N* = 20
ISS (points)	50	36
Age (years)	29	37
Pelvic binder (prehospital) (%)	100	25
Catecholamines (prehospital) (%)	100	48
Hb (g/dl) at arrival in TRU	4.8	8.2
pH	7.1	7.3
Base excess (mmol/l)	− 12.5	− 5.7

### Outcome

Compared to the PVI group of the local Level I Trauma Centre, those patients who died in hospital presented a significantly lower mean Hb of only 5.8 g/dl (vs. 8.2 g/dl), a higher median base deficit of 12.5 mmol/l (vs. 5.7 mmol/l), a younger age of 29 years (vs. 37 years) and a higher ISS of 50 points (vs. 36 points) (Table 4).

### The pelvic vascular injury score (P-VIS)

**Fig. 3:**
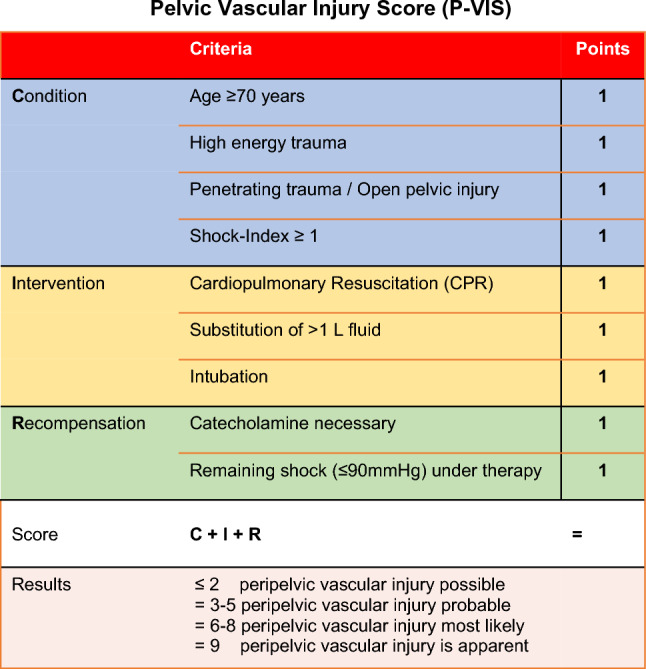
Pelvic vascular injury score (P-VIS) and its validated scoring system

After the analysis epidemiology, therapy and outcome, predictive factors for the presence of peripelvic vascular injury were identified. To detect peripelvic vascular injuries in patients with severe pelvic fractures (AIS ≥ 3) during the prehospital trauma management, predictors that can potentially define a score were identified of the data of the Level I Trauma Centre. In a second step, these predictive factors were further refined within the data of the TR-DGU. After that process, nine predictive factors were identified and incorporated into the peripelvic vascular injury score (P-VIS) to be applied in the prehospital setting, without using any technical devices (Table 5, Fig. 3):

**Table 5:** The nine predictive factors for peripelvic vascular injury, defining the P-VIS

Condition of the patient	Intervention	Recompensation
Age ≥ 70 years	Cardio-pulmonary-resuscitation (CPR)	Necessity of catecholamine substitution
High-energy trauma	Substitution of > 1 l fluid	Remaining shock (RRsys ≤ 90 mmHg) under therapy
Penetrating trauma/open pelvic injury	Intubation	
Shock Index ≥ 1		

Condition of the patient:

- Age ≥ 70 years
- High-energy-trauma
- Penetrating trauma/open pelvic injury
- Shock Index ≥ 1

Pre-hospital interventions:

- Cardio-pulmonary-resuscitation (CPR)
- Substitution of > 1 l fluid
- Intubation

Recompensation:

- Necessity of catecholamine substitution
- Remaining shock (≤ 90 mmHg) under therapy

**Fig. 4:**
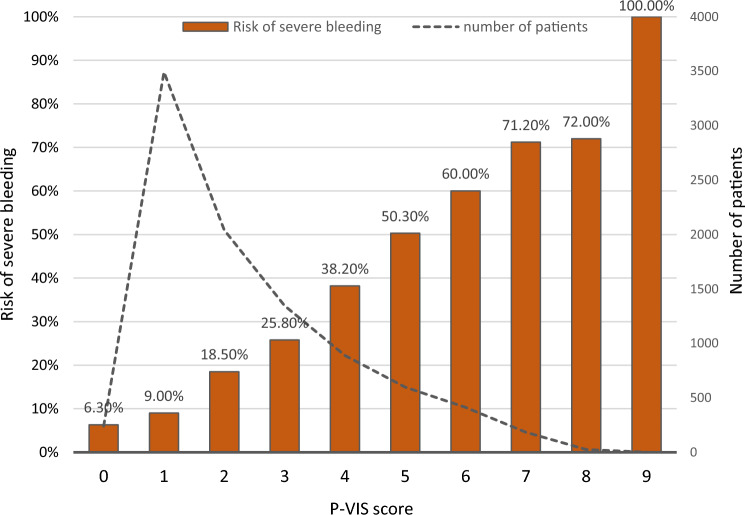
Distribution of patients (*N*) within the P-VIS results (line) and percentage of the presence (positive predictive value, ppv) of a peripelvic vascular injury within the P-VIS results (column). Data of the TR-DGU data set (*N* = 9227)

The process of refining the score within the TR-DGU included 9227 patients with unstable pelvic fractures (Fig. 1). After defining single items as predictive parameters and comparing PVI_TR-DGU_ (*N* = 2090) to C_TR-DGU_ (*N* = 7137) (Table 6), a multivariate logistic regression analysis was performed. To define the score, those predictors which showed an odds ratio (OR) between 1.5 and 2.0 (Table 7) were nominated score 1 point within the P-VIS. The “low fall” injury mechanism showed a reduced risk for PVI_TR-DGU_ (OR 0.51), compared to all other mechanisms. Therefore, the item “high energy trauma” representing all other mechanisms than low falls was included into the P-VIS. Figure 4 shows the distribution of the score in the study population (*N* = 9227) and presents the score results with the prevalence of vascular injury.

**Table 6:** Comparison of C_TR-DGU_ and PVI_TR-DGU_ of the TR-DGU data set, identifying the predictive parameters

	Control (C_TR-DGU_) (*N* = 7137)	PVI_TR-DGU_ (*N* = 2090)
Demographics, mechanism of accident, med. status, prehospital procedures		
Age (years), mean (SD)	48.6 (20.9)	50.5 (21.0)
Age ≥ 70 years, (*n*)	1351 (18.9%)	475 (22.8%)
Sex (male), (*n*)	4524 (63.4%)	1362 (65.2%)
Penetrating mechanism, (*n*)	80 (1.1%)	62 (3.0%)
Motor vehicle accident, (*n*)	1516 (22%)	377 (18%)
Motorcycle accident, (*n*)	1032 (15%)	373 (18%)
Bicycle accident, (*n*)	427 (6.1%)	121 (5.8%)
Pedestrian, (*n*)	857 (12.1%)	337 (16.3%)
Traffic accident, overall, (*n*)	3997 (56%)	1275 (61%)
High fall, (*n*)	2103 (30%)	625 (30%)
Low fall, (*n*)	617 (8.7%)	68 (3.3%)
Injury Severity Score (ISS), mean (SD)	25.4 (11.9)	44.8 (14.3)
Systolic blood pressure (BP) (mmHg), mean (SD)	124 (30)	100 (41)
BP ≤ 90 (mmHg), (*n*)	959 (13.4%)	845 (40.4%)
Heartrate (B/min), mean (SD)	92 (22)	97 (34)
Shock Index, mean (SD)	0.80 (0.46)	1.09 (0.99)
Shock Index ≥ 1, (*n*)	1307 (18.9%)	893 (47.3%)
GCS 3–8, (*n*)	940 (13.6%)	728 (35.9%)
Intubation, (*n*)	1832 (25.7%)	1191 (57.0%)
Catecholamine application, (*n*)	671 (9.4%)	696 (33.3%)
Chest tube, (*n*)	258 (3.6%)	252 (12.1%)
Cardiopulmonary Resusc. (CPR), (*n*)	118 (1.7%)	258 (12.3%)
≥ 1000 ml fluid, (*n*)	1381 (19.3%)	845 (40.4%)
Status at admission, outcome		
Blood transfusion, (*n*)	993 (4.0%)	1428 (69.0%)
Coagulopathy at admission, (*n*)	963 (14.2%)	938 (49.2%)
FAST positive, (*n*)	392 (9.4%)	315 (28.8%)
Massive transfusion (10 + units), (*n*)	–	517 (25%)
Early transfer out (< 48 h), (*n*)	233 (3.3%)	43 (2.1%)
In-hospital mortality (no early transfer out), (*n*)	573 (8.3%)	759 (37.1%)
Length of hospital stay (day, MV/median; no early transfer out)		
Survivor	24.6/20 (*N* = 6330)	43.1/35 (*N* = 1287)
Non-survivor	8.8/3 (*N* = 571)	6.3/1 (*N* = 754)

**Table 7:** Defining the predictive parameters regarding regressions coefficient, significance (*p* value < 0.05), odds ratio (OR) and confidence interval

	Regressions coefficient	Standard deviation	*p* value	Odds ratio (OR)	95% confidence interval for OR
Age ≥ 70 years	0.465	0.069	< 0.001	1.59	1.39–1.82
Male	0.073	0.058	0.209	1.08	0.96–1.21
Penetrating injury	0.667	0.192	< 0.001	1.95	1.34–2.84
Traffic accident	0.011	0.120	0.925	1.01	0.80–1.28
High fall > 3 m	0.037	0.125	0.769	1.04	0.81–1.32
Low fall < 3 m	− 0.672	0.179	< 0.001	0.51	0.36–0.73
BP ≤ 90 mmHg	0.442	0.091	< 0.001	1.56	1.30–1.86
Intubation	0.515	0.075	< 0.001	1.67	1.45–1.94
Catecholamine	0.484	0.080	< 0.001	1.62	1.39–1.90
Chest tube	0.175	0.107	0.102	1.19	0.97–1.47
CPR	0.731	0.133	< 0.001	2.08	1.60–2.70
Volume > 1 l	0.443	0.063	< 0.001	1.56	1.38–1.76
Shock Index ≥ 1	0.541	0.082	< 0.001	1.72	1.46–2.02
GCS ≤ 8	0.189	0.078	0.016	1.21	1.04–1.41

A score of ≤ 2 points represents a low risk of a vascular injury (10%), 3–5 points represent a moderate risk for peripelvic vascular injury (20–50%), and with 6 or more points a peripelvic vascular injury is most likely (> 50%).

**Fig. 5:**
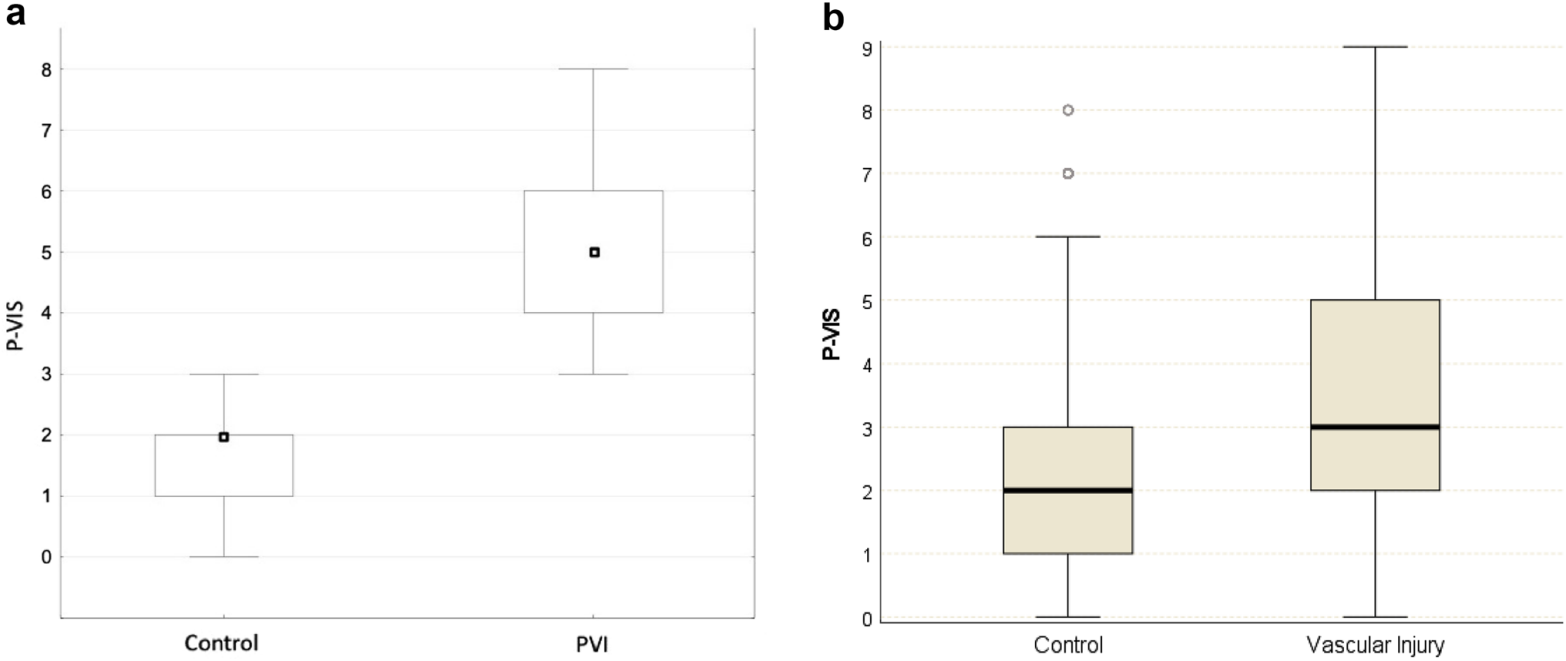
**a** Box-Plot of P-VIS-scoring in PVI and control cohort. PVI: median 5 points; control without peripelvic vascular injury: Median 2 points (*p* < 0.0001). **b** Box-Plot of P-VIS-scoring in PVI_TR-DGU_ and Control_TR-DGU_. PVI_TR-DGU_: median 3 points; Control_TR-DGU_ without peripelvic vascular injury: median 2 points (*p* < 0.001)

Applying the final score to the data set of the TR-DGU, PVI_TR-DGU_ showed a median score of 3 (interquartile range 0–9) points and Control_TR-DGU_ a median of 2 points (interquartile range 0–6) (*p* < 0.001, Fig. 5b).

**Fig. 6:**
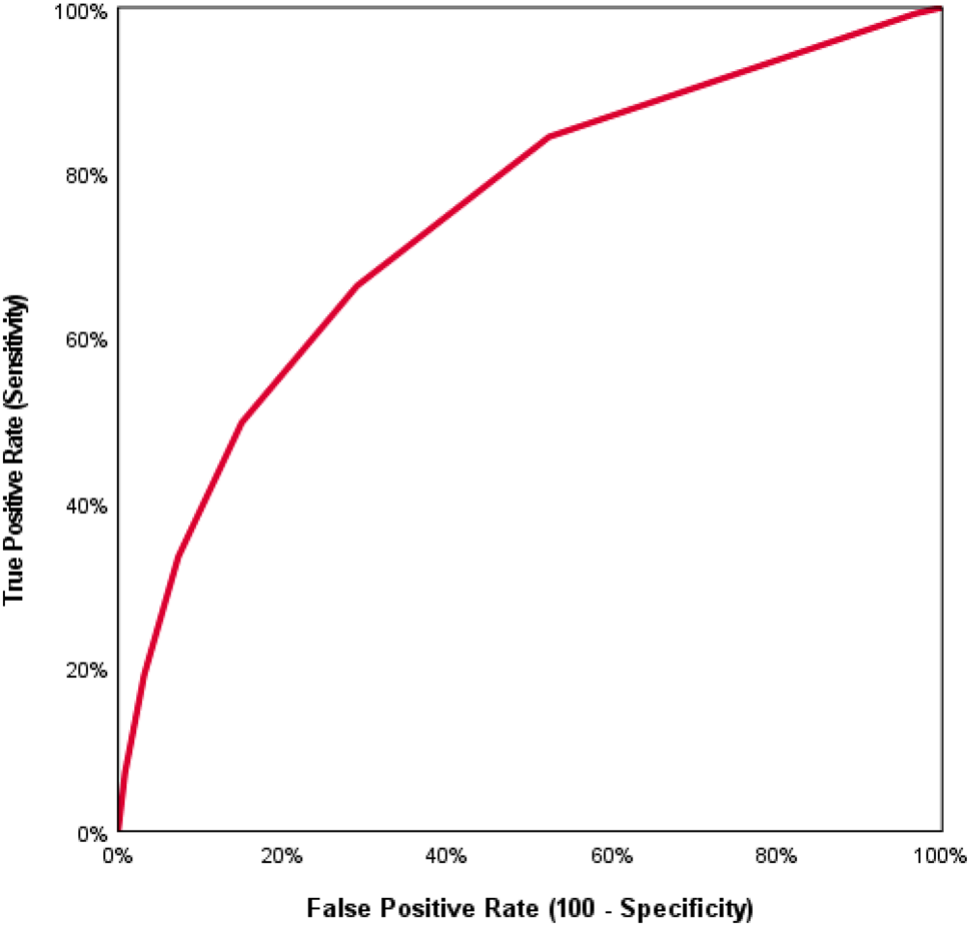
ROC curve (receiver operating characteristic curve) with AUC (area under the curve) = 0.74 (CI-95 0.73–0.76)

The area under the ROC curve (receiver operating characteristic curve) was 0.74 (95% CI 0.73–0.76). Figure 6 presents the performance of the score by plotting the true positive rate (TPR; sensitivity) against the false positive rate (FPR; 100—specificity) at various thresholds.

The odds ratio for peripelvic vascular injury was 24.3 for the patients who scored ≥ 3 points when compared with the patients who scored ≤ 2 points.

When applying the initial score to the study population of the Level I Trauma Centre (*N* = 467), it revealed a median score of 5 points (interquartile range 3–8; 75% with 6 + points) in patients with peripelvic vascular injury and for those without a relevant bleeding the median score was 2 points (interquartile range 0–3; *p* < 0.001, Fig. 5a).

## Discussion

This study confirms that pelvic fractures with concomitant vascular injury are rare yet life-threatening events. It reinforces the importance of this topic though, by showing that vascular injuries in combination with pelvic fractures increases the mortality rate significantly. The challenge was to develop an instrument to detect vascular injury in pelvic trauma patients at an early stage in trauma management and to discuss the question if early detection and treatment of vascular injury can make a difference with respect to outcome.

In this study, 5.1% of patients with severe injuries (ISS ≥ 16) and significant pelvic fractures (AIS ≥ 3) suffered a peripelvic vascular injury. This is concordant to other studies [10, 13, 15, 16]. The results of the TR-DGU even show a percentage of 7.6% severe pelvic fractures (AIS ≥ 3) of all severely injured within a 10-year time period and 22.7% of the severe pelvic injuries show significant peripelvic vascular injuries. Leading to the assumption that peripelvic vascular injuries are an even more important concomitant injury in severe pelvic fractures than described so far.

The data also indicate that patients with pelvic trauma and vascular injury show a significantly higher mortality rate (17.4%) compared to pelvic trauma without vascular injury (10.3%). Other studies support these findings [3, 7, 9, 12, 13, 16].

Whilst WBCT in the TRU provides most of the information needed to answer the question on how severe the pelvic and peripelvic injury is, clinical findings in the prehospital setting only rely on the trauma team’s ability to detect the pattern of injury by physical examination and interpreting the haemodynamic and neurological status of the patient. During the prehospital management, the diagnosis of an unstable pelvic fracture is based solely on clinical examination and signs of hypovolemia in case of severe bleeding. The value of clinical examination of a mechanical instability of the pelvis has been discussed intensively. Most clinical guidelines still suggest to perform a clinical examination of the stability of the pelvis [16, 17]. The sensitivity of this examination has been found as low as 53% [24]. The positive predictive value of the examination is quite high which means that if a mechanically unstable pelvis was found, the diagnosis of a pelvic fracture would be probable. Thus, it does not provide any information about vascular injury, which can only be assumed by the grade of instability of the pelvis and the overall status of the patient.

Haemodynamic instability is defined as a systolic blood pressure < 90 mmHg. This criterion detected two-third of the patients with pelvic fracture (AIS_Pelvis_ ≥ 3) and significant vascular injury (PVI). At an early stage in young patients, this criterion though can be negative due to the individual physiological reserve and capacity to compensate.

The authors therefore developed a clinical score to identify patients with pelvic fracture being at risk of a concomitant significant vascular injury. It relies purely on clinical findings that can easily be obtained in the prehospital setting. In patients with a score of 0–2 points, it represents almost no risk of a vascular injury, 3–5 points a peripelvic vascular injury being probable, 6–8 points identifies a peripelvic vascular injury being most likely and 9 points represents an apparent vascular injury. This score is easy to apply and would result in early stabilisation of the pelvis by applying a pelvic binder as well as calculated resuscitation, immediate transfer to a Level I Trauma Centre and activation of an extended trauma team including (endo)vascular repair capacity and high volume of blood transfusion.

Interesting enough the underlying data of the present study revealed that only 25% of the patients with pelvic fracture (AIS_Pelvis_ ≥ 3) and significant vascular injury had a pelvic binder being applied when arriving in the TRU. This study is no proof that the application of a pelvic binder would have made a difference in the patient’s outcome. But the available evidence suggests that in case of a pelvic fracture with significant bleeding, reduction of the intra-pelvic volume is helpful to reduce the bleeding [7, 9–12, 15–17]. Other studies showed that time to control the bleeding is of the essence [4, 16, 24, 25]. That is why there is a need for an easy to use prehospital score to support the prehospital decision-making and accelerate the further management to an optimised trauma care.

There is no clinical study with available proof that early detection of patients with pelvic fractures and significant peripelvic vascular injury improves survival rates. But all available studies on severely injured patients with significant bleeding suggest that time is crucial and maximum care is needed as early as possible [3–7, 9, 15–19, 24, 25].

## Conclusion

The pelvic vascular injury score (P-VIS) allows an initial risk assessment for the presence of a vascular injury in patients with unstable pelvic injury. Thus, the management of these patients can be positively influenced at a very early stage. If the P-VIS scores ≥ 3 points, a pelvic sling, tranexamic acid and resuscitation in a calculated manner with a permissive hypotension should be applied. If the P-VIS scores ≥ 6 points, the allocation of the patient to a Level I Trauma Centre with 24/7 capability of an interdisciplinary trauma team should be done on scene immediately. The surgical and/or the endovascular repair capacity and high level intensive care as well as the availability of enough blood products for the advanced resuscitation and the initiation of a massive transfusion protocol should be prepared.

## Limitations

Several limitations were noticed and deserve mention. First, the retrospective study design has already been mentioned. A prospective multi-centre study design is needed due to the rare event of this severe injury.

Second, due to limited diagnostics in the prehospital setting, the score needs to be safe and easy to apply with only limited information. This is why it might look like a general bleeding score more than a specific score for pelvic fractures. The statistics though are limited to the existence of a pelvic fracture. It cannot be used to calculate the risk of a general trauma associated bleeding.

Third, no data exist so far regarding long-term outcome proof that an immediate transfer to a Level I Trauma Centre and the application of a pelvic binder is the main life-saving procedure.

This study was designed to identify surrogate parameters for the risk of the presence of a severe peri-pelvic vascular injury in pelvic fracture patients and to incorporate those factors into a pelvic vascular injury score. In addition to the prehospital assessment of the individual pattern of injury and several scoring systems that help decision-making on scene already, this score provides an opportunity to assess the risk of a rare yet life-threatening injury.

## Data Availability

Data sharing not applicable to this article as no datasets were generated or analysed during the current study.
